# Recall and Interviewer Bias in Parental Report of Pediatric Exposure to Aromatic Plant Ingredients in Personal Care Products: Development and Validation of a More Accurate Approach

**DOI:** 10.1155/2021/9924621

**Published:** 2021-08-23

**Authors:** Jessie Hawkins, Christy Hires, Elizabeth Dunne, Lindsey Keenan

**Affiliations:** Franklin Health Research Foundation, Franklin, TN 37067, USA

## Abstract

Lavender and tea tree essential oils are traditionally considered to be mild, gentle, and safe for pediatric populations and are ubiquitous in personal care products. Recent case reports have proposed a potential association between exposure to these ingredients and endocrine disruption, but these reports contain misclassification bias. The purpose of this study is to develop a reliable and valid measurement instrument for the accurate classification of exposure to aromatic plant ingredients in personal care products to be used in epidemiological studies. This study tested the Aromatic Plant Ingredients and Child Health Survey (APICHS) for validity and reliability, contrasting it with the current approach used in clinician's offices. The APICHS was found to have exceptional sensitivity and specificity (100% and 92.86%, respectively) with a positive predictive value of 97.22%, far exceeding the sensitivity and specificity of the method currently in use. The APICHS is a valid, reliable tool for accurate classification of exposure to aromatic plant ingredients in personal care products and should be used for the avoidance of misclassification.

## 1. Introduction

Lavender and tea tree essential oils are among the most common ingredients in personal care products, especially for children [1, 2]. The oils are traditionally considered to be mild, gentle, and safe for pediatric populations [3–5]. Recent sales of personal care products confirm that these are desirable ingredients in household products for families [6]. However, recent case reports have proposed that these two oils are associated with endocrine disruption in children [7]. Due to a lack of epidemiological evidence, experts have advised caution until the proposed link between ingredients and the rare outcome of prepubertal gynecomastia can be investigated. Parents of young children have expressed concern over the lack of clarity on the potential risk posed by these oils, further confirming the urgent need for this epidemiological research.

Because lavender essential oil and tea tree essential oil are ubiquitous in personal care products, identifying and properly classifying exposure status presents a significant challenge. This challenge poses a barrier to the epidemiological research required to scientifically assess the claims that these essential oils act as endocrine disrupting agents in the developing human body.

Lavender and tea tree essential oils do not leave traces at any detectable level within the human body [8, 9]. Key chemicals in these plant extracts have half-lives as short as 14–18 minutes, rendering a laboratory test for past exposure infeasible [10, 11]. To identify historical exposures to these ingredients, many clinicians simply ask parents during an office visit whether or not their child has used any personal care products which contain lavender or tea tree essential oils. This recall question is the only tool utilized to classify the child as exposed or unexposed.

This approach lacks the scientific rigor required to classify exposure status of a child, due to its failure to address information bias, specifically recall bias and interviewer bias, ultimately resulting in misclassification bias [12, 13]. Parents are rarely able to recite the full list of ingredients in all of their child's skincare products, and the stress of a specialist visit with a young child inhibits recall ability, particularly when a diagnosed illness is involved [14, 15].

Similarly, clinicians are not unbiased data collectors; their attitude about the ingredient in question is conveyed to the parent through nonverbal communication. This can result in the Hawthorne effect, when parents answer as they believe they are being led to answer rather than with factual information [16]. Additionally, interviewer bias (when clinicians obtain skewed data in support of preconceived notions) is introduced when clinicians ask cases about exposure status but fail to question noncases about potential exposure [17–19]. Ultimately, this approach produces data which reflects clinician perceptions rather than reality.

In epidemiological studies, misclassification bias, the failure to accurately classify exposed and unexposed patients, distorts research findings. Differential misclassification is particularly problematic because overclassification of exposed cases biases the risk, odds, or rate ratios away from the null [20]. This inaccuracy in data collection leads to an artificially inflated sense of risk.

Because some chemical research has proposed a relationship between these two ingredients and endocrine disruption in children, a valid and reliable method of classifying patients as exposed and unexposed is urgently required [7]. Misclassification has already occurred in the scientific literature regarding these ingredients, with at least one highly publicized case series failing to accurately classify exposures [21]. Misclassification of exposures results in inaccurate reports in the literature and ultimately, the prolonging of key public health issues. Until exposure status can be accurately classified, the epidemiological research studies necessary to investigate this proposed link cannot be conducted.

### 1.1. Objective

The primary purpose of this study is to evaluate the Aromatic Plant Ingredients and Child Health Survey (APICHS) for validity and reliability as a measurement instrument. The purpose of this instrument is to accurately classify children who were exposed to lavender or tea tree essential oil through personal care products and household exposures between the ages of 2 and 15 years old. The secondary objective of this study is to evaluate the prior approach of parental recall for validity and reliability and determine which approach has greater positive predictive value. This study is reported using the STARD checklist for diagnostic accuracy studies.

## 2. Methods

### 2.1. Study Design

This is a prospective study evaluating two methods of measuring pediatric exposure to aromatic ingredients in personal care and household products.

### 2.2. Participants

The population for this study consists of parents of children ages 2–15 years old who live in the United States. Parents were recruited from across the country. Informed consent and instructions for the survey were administered online. After signing the informed consent, the parents completed the health outcome survey and identified personal care products to which their child had been exposed. The study was approved by an independent IRB prior to recruiting the first participants.

### 2.3. Sample Size

A power analysis was conducted using G^*∗*^Power 3.9.1.6 to determine the minimum sample size required to identify a strong correlation (0.8 or higher). Considering 0.05 as statistically significant, with 90% power, a minimum of 23 participants were required to identify a correlation of 0.8 or higher. To account for dropouts and to allow for subgroup analyses, the minimum sample was increased by 50% to 35.

### 2.4. Test Methods

Two methods of measuring exposure were used for this study. The Aromatic Plant Ingredients and Child Health Survey (APICHS) was used, as well as the current approach of a single yes/no question, “Do products used on this child contain (lavender/tea tree) essential oil?” The APICHS was developed specifically for use with this population to improve classification of exposures for both diagnostic and epidemiological research purposes. To ensure that the APICHS did not influence responses to the yes/no question, the APICHS was given after parents responded to the current approach.

### 2.5. Recall Bias

Recall bias is a considerable issue with retrospective measurement and self-reporting, so the APICHS was developed with the understanding that it is impractical to expect parents to recall the full ingredient lists of all items their children have used over the last 2–15 years. Rather than ask parents about ingredients, the instrument uses visual prompts to collect exposure data based on market-ready product usage. Studies have shown that visual prompts are highly effective in promoting memory recall [22–24]. The APICHS uses visual cues with introductory texts to help parents recall which products their child has used.

### 2.6. APICHS Development

The survey contains two sections. Section one includes demographic, health history, health outcomes, and essential oil home use questions. These questions are categorical or dichotomous variables. Section two contains personal care product identification with over 400 images of personal care products, each which either contain one of the essential oils in question or are marketed in such a way to imply that they contain one of the essential oils in question.

To ensure a fully representative population of products which may contain lavender or tea tree essential oils, a comprehensive search across web-based stores, manufacturers, and brick-and-mortar stores was conducted. In addition to individual retailers and web searches, databases such as the Environmental Working Group's Skin Deep database were searched, as well as mass market retailers such as Amazon.com. To capture discontinued products, Internet archives were used as well. When there was more than one formulation for a product, both products were included, along with the visual identifiers. In total, ingredient lists for approximately 4,500 products were evaluated for potential inclusion. If a product implied that it had lavender essential oil, tea tree essential oil, lavender extract, or fragrances, it was included in the screening.

Each product was classified as a wash-off personal care product (i.e., shampoo or body wash), a leave-on product (i.e., lotion, hair styling gel), or an environmental household exposure (i.e., laundry soap). Products were classified as containing pure lavender essential oil, pure tea tree essential oil, primary chemicals found in lavender essential oil (i.e., linalool), primary chemicals found in tea tree oil, or none of the ingredients in question. These allowed for the development of a codebook which classified each suspected exposure as a true exposure or not. The codebook was based on the product's ingredient list, which is subject to FDA cosmetic regulations [25].

### 2.7. Analysis

Because direct observation is not possible and there is no existing measurement with which to compare, validity and reliability were evaluated through test-retest with one week between responses. Because one of the greatest threats to retrospective data collection is recall bias, test-retest reliability serves as an optimal measure to evaluate the appropriateness of this instrument for the purpose of diagnostics and epidemiological research on the risks and safety of exposures to ingredients in personal care products.

Using the index test, total exposure scores were calculated at point 1 and point 2 for both lavender and tea tree oil. This provided a figure for the total number of different exposures to each of these ingredients. The mean difference in exposure scores between the two timepoints was computed to evaluate the difference between testing and retesting [26]. These continuous outcomes were evaluated using Pearson's correlation coefficient as well as Lin's concordance correlation coefficient with a *z*-transformation and a bias correction factor [27]. A scatterplot of exposure scores with a line of linear fit was created to produce a visual assessment of the relationship between testing points and a Bland–Altman plot was constructed to identify the limits of agreement between the two timepoints with a 95% confidence interval [28].

To assess diagnostic accuracy, the continuous variable of total exposure was converted to a dichotomous variable of exposed and unexposed. Additionally, the current yes/no single-question approach was assessed. For each measurement test, a Kappa coefficient of agreement was calculated. Diagnostic utility was evaluated via sensitivity, specificity, and positive and negative predictive values with baseline scores as the reference.

## 3. Results

### 3.1. Participant Flow

Of the 70 parents of children aged 2–15 who were recruited to participate, 58 completed the first survey. A total of 53 completed the second survey, one week later. A total of 4 participants failed to completely finish one or both surveys, producing a total sample size of 49. Parents spent an average of 14 minutes on the first survey and 11 minutes on the second survey. The mean age of children represented in the survey was 7.63 (SD = 3.78). There were slightly more females than males (22 or 44.9% and 27 or 55.1%), and 43 (87.76%) of the children were white. Household income and parental education were approximately normally distributed.

### 3.2. Correlation of Total Exposure Values

Lavender exposure scores during the first survey ranged from 0 to 11 products with a mean of 3.20 (SD = 2.86). Exposure scores during the retest survey also ranged from 0 to 11 products with a mean of 3.39 (SD = 3.05). The scatterplot of scores comparing the two measurements, with a line of linear fit demonstrating high levels of agreement. This was confirmed with Pearson's correlation coefficient, which identified a strong linear correlation between the two measures, *r* = 0.821, *n* = 49, *p*=<0.001.

Tea tree exposure scores were much lower for both surveys. Scores ranged from 0 to 2 on the first test and 0–3 on the retest. Average scores were 0.27 (SD = 0.53) on the first test and 0.41 (SD = 0.79) on the retest. The scatterplot of scores comparing the two measurements, with a line of linear fit demonstrates acceptable agreement, which was confirmed by Pearson's correlation coefficient of .73 (*n* = 49, *p*=<0.001).

### 3.3. Concurrent Validity

To evaluate the extent to which questionnaire responses one week apart agree on lavender exposure, Lin's concordance correlation coefficient was calculated with a bias transformation factor. Exposure responses from week 1 and week 2 were found to have an acceptable level of concordance.

Linn's concordance correlation coefficient identified agreement between the two scores (*ρ*_c_ = 0.82, 95% confidence limits with *z*-transformation 0.696, 0.891). See Table 1.

Lin's concordance correlation coefficient was also calculated with a bias transformation factor for the outcome of tea tree exposure. Exposure responses from week 1 and week 2 were found to have a lower level of concordance. Linn's concordance correlation coefficient identified agreement between the two scores (*ρ*_c_ = 0.66, 95% confidence limits with *z*-transformation 0.503, 0.778). See Table 2.

### 3.4. Bland–Altman

**Figure 1:**
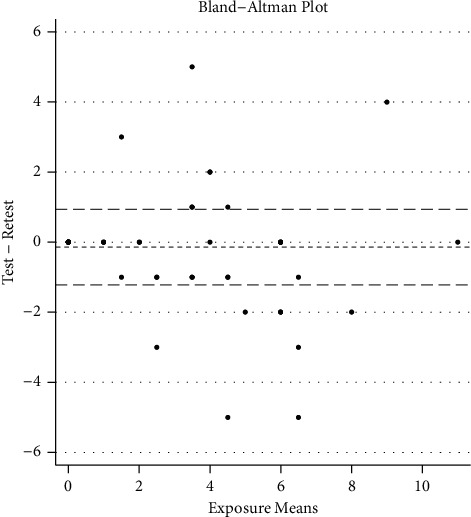
Bland–Altman limits of agreement: −3.194 and 3.725 (95% CI).

A Bland–Altman plot also revealed agreement between responses for total lavender essential oil and total tea tree essential oil exposures. For the lavender essential oil outcome, the mean difference in exposure scores was 0.265 (SD = 1.765), with Bland–Altman limits of agreement of −3.194 and 3.725 (95% CI). On average, responses to exposures were remarkably similar (see Figure 1).

**Figure 2:**
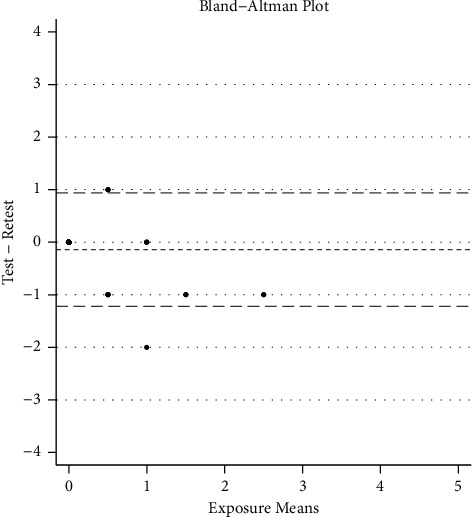
Bland–Altman limits of agreement: −0.916 and 1.201 (95% CI).

For the tea tree essential oil outcome, the mean difference in exposure scores was 0.143 (SD = .540), with Bland–Altman limits of agreement of −0.916 and 1.201 (95% CI) (see Figure 2).

### 3.5. Exposed versus Unexposed

While the continuous outcomes provide utility evaluating the total number of unique exposures, the outcome relevant to most clinicians and epidemiologists is exposure status, regardless of dose. To evaluate the ability of the APICHS method of accurately classifying patients as exposed or unexposed, the variables were converted to dichotomous reflecting complete absence of exposure and any level of exposure. This allowed direct comparison with the current approach of a single yes/no question.

### 3.6. Kappa Coefficient

Cohen's *κ* was performed to determine the extent of agreement between the measures at the two points in time. For the outcome of lavender exposure, the index test produced almost perfect agreement, *κ* = 0.949 (95% CI: 0.850, 1.00), *p*=<0.001), whereas the current approach only produced moderate agreement, *κ* = .550 (95% CI: 0.306, 0.795), *p*=<0.001).

For the outcome of tea tree essential oil exposure, both tests produced moderate agreement, *κ* = 0.609 (95% CI: 0.380, 0.838), *p*=<0.001; *κ* = 0.560 (95% CI: 0.290, 0.829), *p*=<0.001). Fewer than 15 total patients reported tea tree exposure via either method, which could account for the similarity in scores for the outcome of tea tree exposure.

### 3.7. Diagnostic Utility: Sensitivity and Specificity

To evaluate external validity and diagnostic utility, sensitivity, specificity, positive predictive value, and negative predictive value were evaluated. When evaluating exposure to lavender essential oil, sensitivity and specificity were 100% and 92.86%, respectively. Positive predictive value was 97.22% and negative predictive value was 100%, with the model correctly classifying 97.96% of participants. The index test had a 0% false negative rate and a 2.78% false positive rate.

These outcomes were far superior to the current method, which produced sensitivity and specificity of 82% and 75%, respectively. Positive predictive value was 87.10% and negative predictive value was 66.67%, with the model correctly classifying only 79.6% of patients. The current approach had a 33.3% false negative rate and a 12.9% false positive rate.

Similar results were identified on the tea tree exposure analysis. Sensitivity was found to be 61.54%, with 91.67% specificity. Positive predictive value was 73.72% and negative predictive was 86.84%, with the model correctly classifying 83.67% of patients. In contrast the current approach has 73.68% sensitivity and 86.67% specificity, with a 77.78% positive predictive value and 83.87% negative predictive value, correctly classifying 81.63% of exposures.

## 4. Discussion

### 4.1. Limitations

One strength of this approach is that the tool utilizes images, allowing for its use with non-English speaking populations. However, due to variations in consumer product availability, the tool is limited to the United States markets.

Additionally, because new products are introduced to the market on a regular basis, and product formulations are frequently updated, the tool must also be updated at a minimum annually. Given the poor predictive value of the current approach, exposures should be classified using the most accurate measure available, especially given the recent attempts to assign causation based on misclassified data. Inaccurate assignment of causation fails the patient by creating unnecessary burdens and fears of personal care products and also fails to identify the actual cause of endocrine disruption, leaving the patient vulnerable to increased severity.

Another limitation of this study is that the current approach of assessing exposure was conducted without the environmental distress of a pediatrician's office visit or the bias introduced through such settings. These findings do not account for interviewer bias or the Hawthorne effect, so this study likely overestimates the positive predictive value of the current approach to essential oil exposure.

### 4.2. Implications for Practice

The purpose of this study was to develop a reliable and valid instrument for use in epidemiological studies evaluating ingredients in personal care products. The APICHS was developed through a literature review and expert review. It was refined and finalized through a pilot test with retest and psychometric analysis.

Assessments of validity and reliability indicate that the APICHS provides substantial improvement to the current approach, dramatically reducing the risk of misclassification.

The approach is cost-effective, as there is no cost associated with a more thorough approach to identifying exposures of interest, but requires much more time than a single potentially biased question. The tool also requires regular updating to remain current with formulation changes and market trends, requiring clinicians to work with epidemiologists and environmental health specialists to ensure accurate classification of exposures.

## 5. Conclusion

This study provides evidence that the current approach of classification of exposure to aromatic plant ingredients poses a high risk of misclassification through both false positives and false negatives. In contrast, the APICHS was found to have exceptional sensitivity and specificity (100% and 92.86%, respectively) with positive predictive value of 97.22%, far exceeding the sensitivity and specificity of the method currently in use.

Given the widespread use of essential oils in personal care and household products, the potential for endocrine disruption caused by these ingredients is a critical public health issue that needs to be urgently investigated. The APICHS is a valid, reliable tool for accurate classification of exposure to aromatic plant ingredients in personal care products and provides the tools necessary for this important research to be conducted.

## Figures and Tables

**Table 1:** Agreement between lavender exposure measures.

Statistical approach	Statistic	95% confidence interval	*p*
Lin's concordance correlation coefficient *ρ*_*c*_	0.815	0.696, 0.891	<0.001
Pearson's *r*	0.821	—	<0.001
Bias correction factor	0.993	—	—
Bland–Altman's limits of agreement	0.265 (SD = 1.765)	−3.194, 3.725	—

**Table 2:** Agreement between tea tree exposure measures.

Statistical approach	Statistic	95% confidence interval	*p*
Lin's concordance correlation coefficient *ρ*_*c*_	0.662	0.503, 0.778	<0.001
Pearson's *r*	0.731	—	<0.001
Bias correction factor	0.906	—	—
Bland–Altman's limits of agreement	0.143 (SD = 0.54)	−0.916, 1.201	—

## Data Availability

The patient data used to support the findings of this study are restricted by Franklin Health's IRB in order to protect patient privacy. Data are available from Dr. Hawkins, j.hawkins@franklinhealth.org, for researchers who meet the criteria for access to confidential data.
